# Dynamic Ovarian Lymphatic Remodeling Is Associated with Macrophage Accumulation During Follicular Development and Ovulation in Mice

**DOI:** 10.3390/biom16101499

**Published:** 2026-10-15

**Authors:** Chuqing Wu, Dan Chen, Weicheng Tang, Ling Feng, Meng Wu, Shixuan Wang, Yourong Feng

**Affiliations:** 1 Department of Obstetrics and Gynecology, Tongji Hospital, Tongji Medical College, Huazhong University of Science and Technology, Wuhan 430030, China; 2 National Clinical Research Center for Obstetrical and Gynecological Diseases, Wuhan 430030, China; 3 Key Laboratory of Cancer Invasion and Metastasis, Ministry of Education, Wuhan 430030, China

**Keywords:** ovary, lymphatic vessels, follicular development, ovulation, macrophages, CCL21–CCR7 signaling

## Abstract

The ovarian lymphatic vasculature has emerged as a potential regulator of ovarian function, yet its role in follicular development remains poorly defined. Here, we aimed to elucidate the dynamic changes in ovarian lymphatic vessels during follicular development and to determine whether lymphatic vessels contribute to ovarian function by regulating the immune microenvironment. Ovarian lymphatic remodeling was characterized during different follicular stages, throughout the estrous cycle, and following superovulation using immunofluorescence imaging. Lymphangiogenesis was inhibited with the vascular endothelial growth factor receptor 3 (VEGFR3) inhibitor SAR131675 to evaluate the effects on follicular development and macrophage accumulation in mice. Transcriptomic, tissue localization, and migration assays with C-C motif chemokine ligand 21 (CCL21)- or C-C chemokine receptor type 7 (CCR7)- blocking antibodies were used to investigate CCL21-CCR7 signaling in lymphatic endothelial cell (LEC)-mediated macrophage migration. Ovarian lymphatic vessels underwent dynamic remodeling, with lymphatic density peaking during the luteal phase. Macrophages preferentially localized near lymphatic vessels, and their abundance paralleled lymphatic remodeling across reproductive stages. Inhibition of lymphangiogenesis reduced follicular development, ovulation, corpus luteum formation, and macrophage accumulation. Mechanistically, ovarian lymphatic endothelial cells highly expressed CCL21, which promoted macrophage migration through CCR7 signaling. Collectively, these findings identify ovarian lymphatic vessels as previously unrecognized regulators of the ovarian immune microenvironment, with CCL21-CCR7 signaling mediating lymphatic endothelial cell–macrophage communication to support ovarian function.

## 1. Introduction

The ovary is a highly dynamic reproductive and endocrine organ that undergoes continuous structural and functional remodeling throughout the reproductive lifespan [1]. These processes rely on a tightly regulated microenvironment in which blood and lymphatic vessels, immune cells, and extracellular matrix coordinate nutrient and metabolite exchange, steroidogenesis, and cellular communication [2,3]. Ovarian blood vessels have been extensively studied for their roles in supporting follicular growth and steroidogenesis [4,5], with coordinated endothelial proliferation and migration enabling vascular adaptation to the changing demands of the ovary [6,7]. In contrast, the roles of ovarian lymphatic vessels in these processes and their interactions with the local microenvironment remain poorly understood.

Previous studies have characterized the postnatal development and hormonal regulation of the ovarian lymphatic vessels [8,9]. Disruption of ovarian lymphangiogenesis through vascular endothelial growth factor receptor 3 (VEGFR3) blockade impairs follicular maturation and subsequent pregnancy outcomes, suggesting an important contribution of ovarian lymphatic vessels to reproductive function [10]. Accumulating evidence indicates that lymphatic vessels not only serve as conduits for fluid and macromolecule transport but also actively regulate local cellular interactions. However, how lymphatic vessels regulate ovarian function remains to be further investigated.

Lymphatic endothelial cells (LECs) regulate immune surveillance and cell trafficking through the production of chemokines, cytokines, and other regulatory factors [11,12]. Their roles in promoting tissue regeneration, maintaining local cellular niches and facilitating tissue repair after injury have also been demonstrated in bone and intestine [13,14]. In addition, LEC-derived chemokines promote macrophage recruitment during LPS-induced inflammation [15], whereas decidual LEC-derived cytokine (GM-CSF) promotes proinflammatory macrophage polarization in pre-eclampsia [16]. Within the ovary, macrophages are highly plastic innate immune cells with essential roles in follicular development, ovulation, and corpus luteum formation [17]. Yet, the local signals governing their recruitment and spatial organization remain poorly defined. Together, these findings raise the question of whether ovarian lymphatic vessels undergo coordinated remodeling to support follicular development and ovulation through interactions with macrophages.

In this study, we found that ovarian lymphatic networks undergo dynamic remodeling during follicular development, ovulation, and corpus luteum formation, accompanied by corresponding changes in macrophage abundance and distribution. Functionally, local VEGFR3 inhibition reduced ovarian lymphatic expansion and macrophage accumulation, together with fewer corpora lutea and a lower yield of ovulated oocytes. Mechanistically, ovarian LECs exhibited enriched C-C motif chemokine ligand 21 (CCL21) expression, and promoted macrophage chemotaxis through CCL21-CCR7 signaling. Therefore, this study aimed to investigate the dynamic remodeling of ovarian lymphatic vessels and to explore their potential interactions with macrophages in regulating ovarian function.

## 2. Materials and Methods

### 2.1. Animals

All mice used in vivo experiments were purchased from BIONT Biotechnology Co., Ltd. (Wuhan, China) and then housed in ABSL-2 laboratories freely for 1 week for environmental acclimation. Female C57BL/6 mice of different ages were used according to the experimental requirements. Mice that were 10 weeks old were used to investigate ovarian lymphatic and macrophage dynamics during follicular development and across the estrous cycle [18], whereas 6-week-old mice were used for PMSG/hCG-induced superovulation and ovarian lymphangiogenesis inhibition experiments [19,20].

### 2.2. Estrous Cycle and Follicular Development Models

A total of 20 female C57BL/6 mice (10 weeks old) were randomly assigned to four groups (*n* = 5 per group) to evaluate ovarian lymphatic and macrophage dynamics across the estrous cycle. The estrous cycle was monitored by vaginal cytology, and mice were classified into proestrus, estrus, metestrus, or diestrus based on the predominant cell types observed in vaginal smears, as previously described [21]. Ovaries were collected at the corresponding cycle stages for subsequent histological and immunofluorescence analyses.

To evaluate lymphatic and macrophage changes associated with follicular development, ovaries were collected from mice at diestrus, and follicles were subsequently classified into different developmental stages based on their morphological characteristics in ovarian sections. The identified follicular stages included primordial, primary, secondary, antral, and preovulatory follicles, as well as corpora lutea.

### 2.3. Superovulation Models

A total of 20 female C57BL/6 mice (6 weeks old) were randomly assigned to four groups (*n* = 5 per group). Superovulation was induced using a widely established hormonal stimulation regimen, consisting of an intraperitoneal injection of pregnant mare serum gonadotropin (PMSG, 10 IU in 200 μL Solarbio, Beijing, China; P9970) followed by human chorionic gonadotropin (HCG, 10 IU in 200 μL, Aibei, Shanghai, China; M2530) administration 48 h later [22,23]. Ovarian tissues collected 48 h after PMSG administration were defined as the HCG 0 h time point. Mice were sacrificed at 6 h, 12 h, and 18 h after HCG injection by cervical dislocation. Ovaries were carefully dissected from the lower pole of the kidney via a dorsal incision, and surrounding oviducts and adipose tissues were removed under a stereomicroscope. Collected ovarian tissues were either immediately snap-frozen and stored at −80 °C or fixed in 4% paraformaldehyde at room temperature overnight for subsequent paraffin embedding and histological analysis.

### 2.4. Ovarian Lymphangiogenesis Inhibition Models

An additional cohort of 20 female C57BL/6 mice (6 weeks old) was used to establish the ovarian lymphangiogenesis inhibition model. Under a stereomicroscope, intrabursal injections were performed with a NanoFil syringe (WPI, Sarasota, FL, USA; NF33BL-2) equipped with a 33G needle. One ovarian bursa received 5 μL of the VEGFR3 inhibitor SAR131675 (3 mg/mL, 15 μg/ovary; Selleckchem, Houston, TX, USA; No. S2842), prepared in 5% DMSO, 30% PEG300, 5% Tween-80, and 60% double-distilled water, while the contralateral ovary received 5 μL of the same vehicle without SAR131675 using the same injection procedure. Following recovery from anesthesia, mice were randomly assigned to two experimental groups and treated with PMSG (10 IU). At 48 h after PMSG administration, mice in the first group were sacrificed for ovarian tissue collection to evaluate follicular development. In the second group, hCG (10 IU) was administered 48 h after PMSG, and mice were sacrificed 14 h later. Ovaries were collected to evaluate corpus luteum formation, and metaphase II (MII) oocytes were collected from the ampullary region of the oviduct to assess ovulation.

### 2.5. Hematoxylin and Eosin Staining

Paraffin-embedded ovarian sections were deparaffinized in an environmentally friendly dewaxing solution (Servicebio, Wuhan, China; G1128) and rehydrated sequentially through 100%, 95%, 85%, and 75% ethanol. The sections were stained with hematoxylin (Servicebio, G1004), followed by differentiation and bluing, and then counterstained with eosin (Servicebio, G1001) [24]. After staining, the sections were dehydrated through a graded ethanol series, cleared, and mounted with neutral resin (Servicebio, WG10004160). The stained sections were examined under a light microscope, and representative images were acquired under identical imaging conditions.

### 2.6. Ovarian Follicle Counting

The ovaries were serially sectioned and stained with H&E staining, and every section with an 80 μm interval was blindly counted by two sophisticated researchers under the light microscope [25]. Follicles were classified based on established morphological criteria: primordial follicles (PMF) contained an oocyte surrounded by flattened granulosa cells (GCs); primary follicles (PF) consisted of an enlarged oocyte with a single layer of cuboidal GCs; secondary follicles (SF) contained two or more layers of cuboidal GCs; antral follicles (ANF) exhibited fluid-filled cavities; and atretic follicles (ATF) were identified by granulosa cell pyknosis, fragmentation, and disrupted follicular architecture. Only follicles containing a visible oocyte were included in the analysis.

### 2.7. Western Blot

Western Blotting was performed according to standard procedures as previously described [26]. Total protein was extracted from ovarian tissues using RIPA lysis buffer (Servicebio, G2002) supplemented with protease inhibitor cocktail (Servicebio, G2006). Protein concentration was determined using a BCA Protein Assay Kit (Beyotime, Shanghai, China; P0012). Equal amounts of protein (25 μg per sample) were separated by 10% SDS-PAGE and transferred to 0.45 μm PVDF membranes (Millipore, Billerica, MA, USA; IPVH00010). Membranes were blocked with 5% bovine serum albumin (BSA) for 1 h at room temperature and incubated overnight at 4 °C with primary antibodies: iNOS (Abclonal, Wuhan, China; A0312, 1:1000), CCL21 (Abclonal, A1896, 1:1000), CCR7 (Abclonal, A0121, 1:1000), VEGFR3 (Proteintech, Wuhan, China; 220712-1-AP, 1:300), VEGFR2 (Abclonal, A5609, 1:1000), Vinculin (proteintech, 226520-1-AP, 1:10,000), and β-actin (Abclonal, AC026, 1:10,000). The next day, membranes were incubated with HRP-conjugated secondary antibodies for 1 h at 37 °C. Bands were visualized using a WesternBright ECL kit (Advansta, San Jose, CA, USA; K-12045). Band intensities were quantified by densitometric analysis using Image Lab software (Bio-Rad Laboratories, Hercules, CA, USA; version 6.0.1), and the expression levels of target proteins were normalized to the corresponding loading control (β-actin or Vinculin). The normalized protein levels were expressed relative to the control group.

### 2.8. Immunohistochemistry (IHC) and Immunofluorescence (IF)

Immunohistochemistry (IHC) and immunofluorescence (IF) staining were performed on paraffin-embedded and frozen ovarian sections, respectively, according to standard procedures as previously described [27]. Briefly, the ovarian sections were deparaffinized by Environmentally friendly dewaxing clear solution and dehydrated by ethanol gradient, undergoing antigen retrieval, inactivating endogenous catalase, 5% BSA blocking and were incubated with primary antibody LYVE1 (Abcam, Cambridge, UK; ab14917, 1:400), F4-80 (Cell Signaling Technology, Danvers, MA, USA; 70076S, 1:250), CD31 (Invitrogen, Carlsbad, CA, USA; MAB1398Z, 1:100) CCL21 (Immunoway, Suzhou, China; YT5973,1:200; R&D Systems, Minneapolis, MN, USA; MAB4572, 5 µg/mL), CCR7 (R&D Systems, MAB3477, 10 µg/mL), VEGFR3 (proteintech, 220712-1-AP, 1:100), VEGFR2 (Abclonal, A5609, 1: 100) overnight at 4 °C. The sections were incubated with secondary antibodies. Afterwards, the IHC slices were stained with DAB Chromogenic Kit (Servicebio, G1212) and the nuclei were stained with hematoxylin. The IF sections were dripped with DAPI, incubated for 10 min, then mounted with antifade medium (Servicebio, G1401), and photographed under a microscope. For quantitative analysis, IHC and IF staining were quantified using Fiji/ImageJ software (National Institutes of Health, Bethesda, MD, USA; version 2.9.0) and expressed as the percentage of positive staining area or fluorescence-positive area, respectively, relative to the total analyzed tissue area. Images were acquired under identical microscope settings for all experimental groups.

### 2.9. EdU Incorporation and Immunofluorescence Co-Staining

For in vivo proliferation analysis, mice were intraperitoneally injected with EdU (50 mg/kg, Servicebio, G5059) 24 h before euthanasia. EdU incorporation into newly synthesized DNA was detected by a copper-catalyzed azide–alkyne cycloaddition (“click”) reaction [28]. Ovarian tissues were collected, and frozen sections were prepared. Sections were fixed in 4% paraformaldehyde for 15 min at room temperature and washed with PBS. Sections were then permeabilized with permeabilization solution (Servicebio, G1204) for 10 min at room temperature and washed with PBS. After blocking with 10% goat serum (Boster, Wuhan, China; AR0009), sections were incubated with primary antibodies overnight at 4 °C, followed by incubation with fluorescent secondary antibodies for 1 h at 37 °C. After immunofluorescence staining, EdU staining was performed using the Click-iT EdU Alexa Fluor 594 kit according to the manufacturer’s instructions (Servicebio, G1601), with incubation at room temperature for 30 min in the dark. Sections were then washed with PBS and counterstained with Hoechst 33342 for 5 min. Finally, sections were mounted with antifade medium (Servicebio, G1401) and imaged using a fluorescence microscope. For quantification, EdU-positive nuclei and LYVE1-positive cells were identified based on fluorescence signals, and the percentage of EdU-positive cells among LYVE1-positive cells was calculated from randomly selected fields.

### 2.10. Multiplex Immunohistochemistry (mIHC)

Multiplex immunohistochemistry (mIHC) was performed using the Opal™ 7-Color Manual IHC Kit (Akoya Biosciences, Marlborough, MA, USA; NEL861001KT) according to the manufacturer’s instructions, with minor modifications. Paraffin-embedded sections were baked, deparaffinized, and rehydrated as described above. Antigen retrieval was performed using citrate-based antigen retrieval buffer under microwave heating. Briefly, the retrieval solution was heated at high power until boiling and then maintained at medium–low power for two 7 min cycles, with the solution level checked between cycles to ensure adequate tissue coverage. After cooling to room temperature, sections were washed with TBST and blocked with 10% goat serum. Primary antibodies F4/80 (CST, 77699, 1:250), LYVE1 (Abcam, ab14917, 1:400), and CD3 (CST, 78588, 1:400) were sequentially applied for 1 h at 37 °C, followed by incubation with an HRP-conjugated secondary antibody for 10 min at 37 °C. After washing with TBST, the corresponding Opal fluorophore working solution was applied for 10 min at room temperature in the dark for tyramide signal amplification. Sections were then washed with TBST, and antibody complexes were removed under microwave heating as described above before the next staining cycle. The blocking, primary antibody incubation, HRP-conjugated secondary antibody incubation, and Opal fluorophore labeling steps were repeated sequentially for each target antigen. F4/80, LYVE1, and CD3 were detected using Opal 520, Opal 570, and Opal 620 fluorophores, respectively. Following completion of all staining cycles, nuclei were counterstained with DAPI for 5 min. Sections were subsequently washed, mounted with antifade mounting medium, and scanned using a P250 automated slide scanner (3DHISTECH, Budapest, Hungary; FLASH III).

### 2.11. Transwell Migration Assay

Human lymphatic endothelial cells (HLECs, WHELAB, shanghai, China; C1259) were cultured in Endothelial Cell Medium (ECM, ScienCell Research Laboratories, Carlsbad, CA, USA; #1001) supplemented with 10% fetal bovine serum (FBS), endothelial cell growth supplement, and 1% penicillin/streptomycin. SVEC4-10 cells (ATCC, Manassas, VA, USA; CRL-2181), a murine lymphatic endothelial cell line [29], were cultured in high-glucose DMEM supplemented with 10% FBS. When HLECs or SVEC4-10 cells reached approximately 80% confluence, the cells were washed twice with PBS and incubated in serum-free DMEM containing 1% penicillin/streptomycin for 24 h. The conditioned media were collected, passed through a 0.22 μm sterile membrane filter to remove cellular debris, and designated as HLEC-conditioned medium (HLEC-CM) and SVEC4-10-conditioned medium (SVEC4-10-CM), respectively.

For Transwell migration assays, as previously described with minor modification [30], THP-1 cells (Procell, Wuhan, China; CL-0233) were seeded into Transwell inserts with an 8 μm pore size (Greiner Bio-One, Frickenhausen, Germany; 66263) at a density of 5 × 10^4^ cells per insert. THP-1 cells were cultured in RPMI-1640 complete medium supplemented with PMA (MedChemExpress, Shanghai, China; HY-18739) at a final concentration of 100 ng/mL for 24 h to induce adhesion and differentiation toward an M0 macrophage phenotype. After PMA treatment, the medium in the upper chambers was replaced with serum-free DMEM, and 600 μL of medium was added to the lower chamber. The lower chamber was assigned to six groups: (1) control, containing DMEM supplemented with 10% FBS; (2) HLEC-CM supplemented with 10% FBS; (3) HLEC-CM supplemented with 10% FBS and a neutralizing antibody against CCL21 (R&D Systems, MAB4572, 3 μg/mL); (4) HLEC-CM supplemented with 10% FBS and a neutralizing antibody against CCR7 (R&D Systems, MAB3477, 5 μg/mL); (5) DMEM supplemented with 10% FBS and the CCL21 neutralizing antibody (3 μg/mL); and (6) DMEM supplemented with 10% FBS and the CCR7 neutralizing antibody (5 μg/mL). For groups containing neutralizing antibodies, the antibodies were pre-incubated with the corresponding medium for the indicated concentrations before addition to the lower chamber. The cells were incubated for 24 h at 37 °C in a humidified atmosphere containing 5% CO_2_.

After 24 h, the inserts were removed and washed twice with PBS. The membranes were fixed with 4% paraformaldehyde for 10 min and then stained with ready-to-use crystal violet staining solution (Servicebio, G1014) for 10 min. After staining, non-migrated cells on the upper surface of the membrane were gently removed using a cotton swab. The membranes were subsequently washed three times with PBS and allowed to dry. Migrated cells on the lower surface of the membrane were imaged under a microscope, and six randomly selected fields per insert were analyzed using ImageJ software to quantify the number of migrated cells.

To further validate these findings in a murine cell system, RAW264.7 cells were subjected to the same Transwell migration procedure and conditions described above. The lower chamber was similarly divided into six groups: (1) DMEM + 10% FBS; (2) SVEC4-10-CM + 10% FBS; (3) SVEC4-10-CM + 10% FBS + CCL21 neutralizing antibody (3 μg/mL); (4) SVEC4-10-CM + 10% FBS + CCR7 neutralizing antibody (5 μg/mL); (5) DMEM + 10% FBS + CCL21 neutralizing antibody (3 μg/mL); and (6) DMEM + 10% FBS + CCR7 neutralizing antibody (5 μg/mL). All other Transwell procedures, including incubation, fixation, crystal violet staining, imaging, and quantification, were performed as described above.

### 2.12. RNA Extraction and Quantitative Reverse Transcription-PCR

Total RNA was extracted from ovarian tissues or cultured cells using RNAiso Plus (Takara Bio Inc., Kusatsu, Japan, 9109) according to the manufacturer’s instructions. RNA concentration and purity were assessed using a NanoDrop 2000 spectrophotometer (Thermo Fisher Scientific, Waltham, MA, USA). For reverse transcription, 2 μg of total RNA was reverse-transcribed into cDNA using the HiScript II Q RT SuperMix for qPCR (+gDNA wiper) kit (Vazyme, Nanjing, China; R423-01), following the manufacturer’s instructions. Quantitative real-time PCR (RT-qPCR) was performed using Taq Pro Universal SYBR qPCR Master Mix (Vazyme, Q712-02) on a CFX96 Real-Time PCR Detection System (Bio-Rad). Gene-specific primers were used for amplification, and the primer sequences are provided in the table below. Each sample was analyzed in technical replicates. Relative gene expression levels were normalized to GAPDH and calculated using the 2^−ΔΔCt^ method as previously described [31].The sequences of primers used for qRT-PCR are listed in Table 1.

### 2.13. Statistical Analysis

All data are expressed as mean ± SEM unless otherwise indicated. The normality of continuous data was assessed using the Shapiro–Wilk test before statistical analysis. For normally distributed data, comparisons between two independent groups were performed using an unpaired Student’s *t*-test, whereas comparisons among multiple groups were performed using one-way ANOVA followed by Tukey’s multiple comparisons test. For non-normally distributed data, the Mann–Whitney *U* test was used for comparisons between two independent groups, and the Kruskal–Wallis test followed by Dunn’s multiple comparisons test was applied for multiple-group comparisons. For paired comparisons between SAR131675-treated ovaries and the contralateral vehicle-treated ovaries from the same mouse a paired Student’s *t*-test was used for normally distributed data, whereas the Wilcoxon signed-rank test was used for non-normally distributed data. Categorical data were analyzed using the chi-square test. Statistical analyses were performed using SPSS (version 13.0) and GraphPad Prism (version 8.1.2; GraphPad Software, San Diego, CA, USA; version 8.1.2). A two-sided *p* value < 0.05 was considered statistically significant.

### 2.14. Generative AI Use

ChatGPT (GPT-6.0; OpenAI) was used to assist with English-language editing, including grammar, sentence structure, spelling, and formatting. The use of generative AI was limited to language-related assistance and did not involve data analysis, data interpretation, image generation, or image processing. All scientific content and the final manuscript were reviewed and verified by the authors.

## 3. Results

### 3.1. Dynamic Lymphatic Remodeling Accompanies Macrophage Accumulation During Ovarian Follicular Development and Luteal Formation

**Figure 1:**
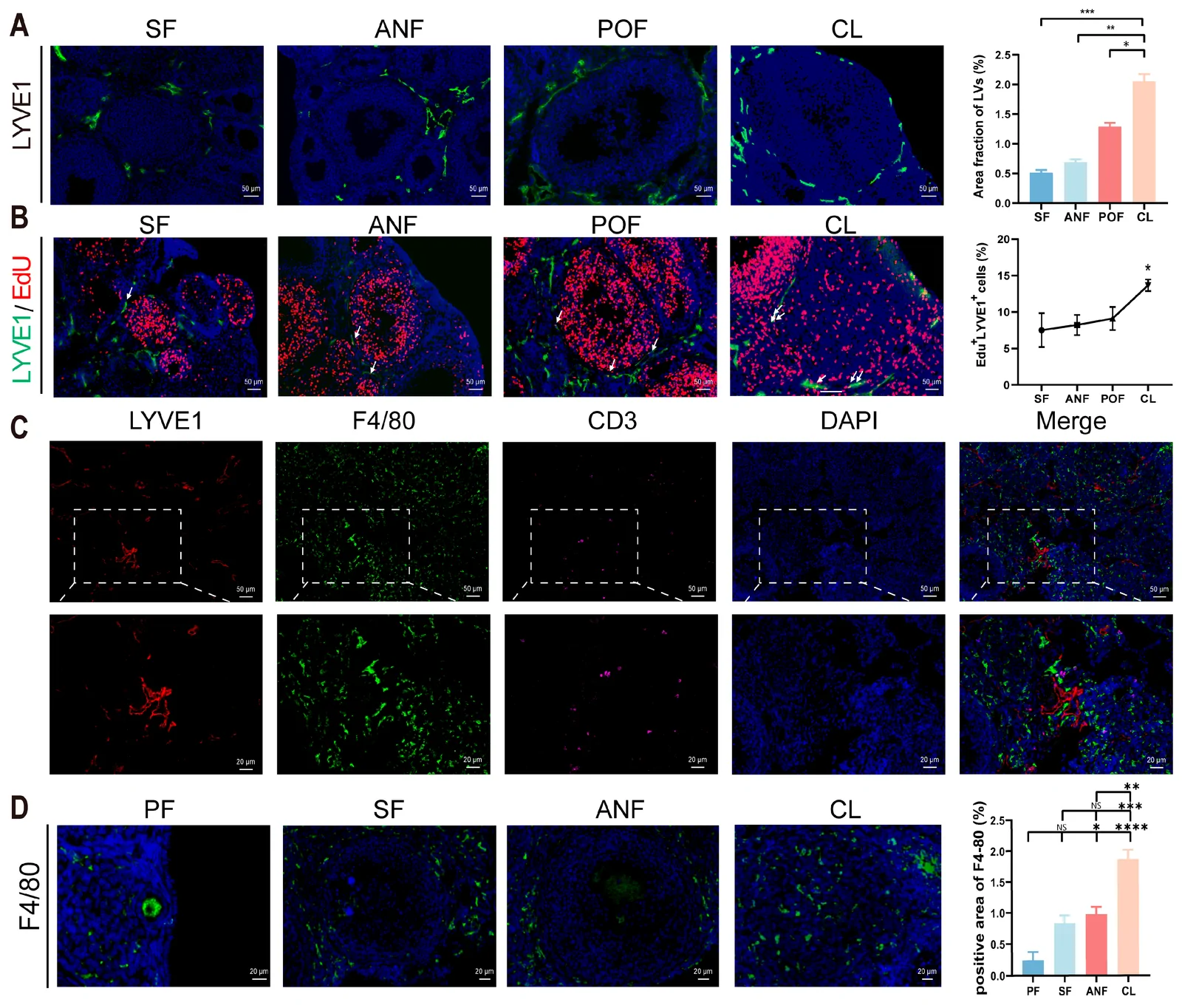
Dynamic remodeling of ovarian lymphatic vessels accompanies macrophage accumulation during follicular development and corpus luteum formation. (**A**) Representative immunofluorescence images of LYVE1^+^ lymphatic vessels (green) in ovarian sections from 10-week-old C57BL/6 mice at the SF, ANF, POF, and CL stages (**left**). Scale bars, 50 µm. Quantification of LYVE1^+^ lymphatic vessel area fraction at different follicular stages (*n* = 6). (**B**) Co-immunofluorescence staining of LYVE1 (green) and EdU (red) showing proliferating LECs at SF, ANF, POF, and CL stages (**left**). Scale bars, 50 µm. White arrows indicate LYVE1^+^EdU^+^ proliferating LECs. The proportion of proliferating LECs was quantified as the percentage of EdU-positive cells among LYVE1-positive cells (*n* = 6). (**C**) Representative multiplex immunohistochemistry images showing the distribution of LYVE1^+^ lymphatic endothelial cells, F4/80^+^ macrophages, and CD3^+^ T lymphocytes in ovarian sections. Scale bars, 50 µm (20× magnification, upper panels) and 20 µm (40× magnification, lower panels). (**D**) Representative images of F4/80^+^ macrophages at the primary follicles (PF), SF, ANF, and CL stages (**left**), Scale bars, 20 µm. Quantification of F4/80^+^ macrophage area fraction at different follicular stages (*n* = 6). Data are presented as the mean ± SEM. Statistical analyses were performed using one-way ANOVA. NS, not significant, * *p* < 0.05, ** *p* < 0.01, *** *p* < 0.001, **** *p* < 0.0001.

To explore lymphatic remodeling during follicular development and corpus luteum formation, we examined lymphatic vessel endothelial hyaluronan receptor 1 (LYVE1) staining in ovaries from 10-week-old mice at sexual maturity. Follicles were categorized into primordial follicles (PMF), primary follicles (PF), secondary follicles (SF), antral follicles (ANF), and preovulatory follicles (POF) based on established morphological criteria and follicle diameter [32]. LYVE1-positive vessels were detected in perifollicular regions, including the thecal compartment, and were prominent around corpora lutea (Figure 1A). The LYVE1-positive area increased across the follicular stages examined and was greatest in corpus luteum-associated regions. LYVE1/EdU co-staining also identified a higher proportion of LYVE1^+^EdU^+^ cells in these regions than around earlier-stage follicles (Figure 1B and Figure S1A). The EdU-negative control was shown in Figure S1F. These observations indicate that ovarian lymphatic vessels and proliferative changes accompany follicular development and early luteal remodeling.

The main immune cells in the ovary are macrophages and T-cells. We next examined the distribution of macrophages and T-cells relative to LYVE1-positive vessels. Multiplex staining showed F4/80-positive macrophages throughout the ovarian stroma and perifollicular regions, with some cells located adjacent to LYVE1-positive structures; CD3-positive T-cells were less frequently observed in the sections examined (Figure 1C). The F4/80-positive area increased across the follicular stages assessed and was highest in corpus luteum-associated regions (Figure 1D). These results suggest that macrophage accumulation is associated with ovarian lymphatic vessels and may participate in the regulation of follicular development and corpus luteum formation.

### 3.2. Dynamic Lymphatic Remodeling Accompanies Macrophage Accumulation Across the Estrous Cycle

**Figure 2:**
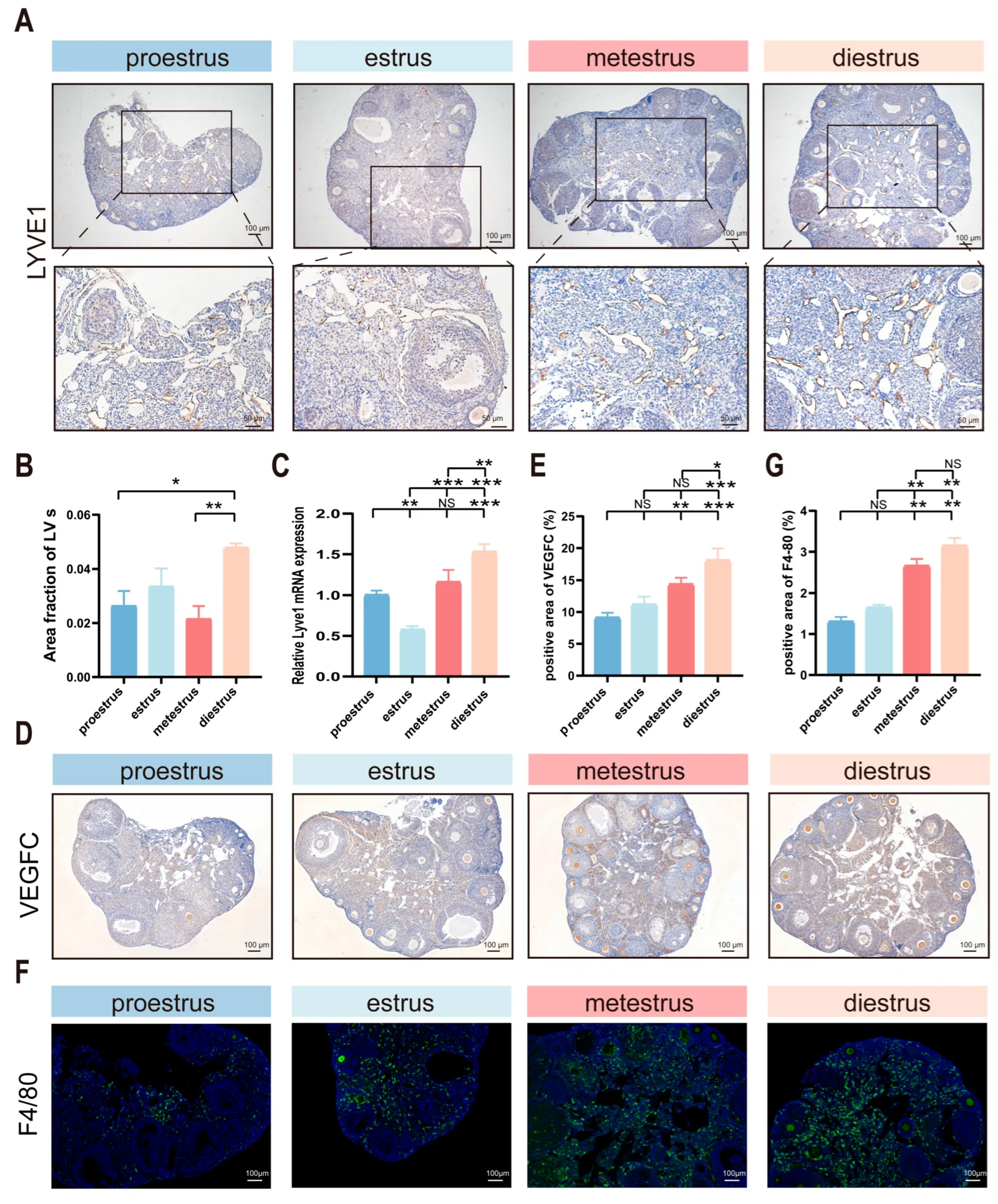
Dynamic remodeling of ovarian lymphatic vessels and macrophage accumulation across the estrous cycle. (**A**) Representative immunohistochemistry images of LYVE1^+^ lymphatic vessels in ovarian sections from 10-week-old C57BL/6 mice at the proestrus, estrus, metestrus, and diestrus stages. Scale bars, 100 µm (10× magnification, upper panels) and 50 µm (20× magnification, lower panels). (**B**) Quantification of LYVE1^+^ lymphatic vessel area at different estrous cycle stages (*n* = 5). (**C**) Relative *Lyve1* mRNA expression levels at different estrous cycle stages (*n* = 3). (**D**) Representative immunohistochemistry images of VEGFC in ovarian sections from 10-week-old C57BL/6 mice at the proestrus, estrus, metestrus, and diestrus cycles. DAB-positive staining indicates VEGFC expression. Scale bars, 100 µm. (**E**) Quantification of VEGFC-positive area fraction at different estrous cycle stages (*n* = 5). (**F**) Representative immunohistochemical images of F4/80^+^ macrophages in ovarian sections at different estrous cycle stages. F4/80 staining is shown in green. Scale bars, 100 µm. (**G**) Quantification of F4/80^+^ macrophage area at different estrous cycle stages (*n* = 5). Data are presented as the mean ± SEM. Statistical analyses were performed using one-way ANOVA. NS, not significant, * *p* < 0.05, ** *p* < 0.01, *** *p* < 0.001.

To determine whether these changes were also evident during the natural estrous cycle, we examined ovaries from 10-week-old mice with regular estrous cycles at proestrus, estrus, metestrus, and diestrus, and found that both the number and area fraction of LYVE1-positive lymphatic vessels were significantly increased during diestrus (Figure 2A,B and Figure S1B). Ovarian *Lyve1* transcript abundance and VEGFC-positive area were also highest at diestrus (Figure 2C–E). In addition, F4/80^+^ macrophage abundance was significantly elevated during metestrus and diestrus (Figure 2F,G and Figure S1C,D). IgG control and negative control images for immunohistochemical staining are shown in Figure S1E. These results indicate that ovarian lymphatic vessels undergo estrous cycle-dependent remodeling, peaking at diestrus, which corresponds to the luteal phase. The concurrent increase in F4/80^+^ macrophages suggests that lymphatic remodeling is temporally coupled with macrophage accumulation, consistent with a role for lymphatic vessels in immune cell recruitment.

### 3.3. Dynamic Lymphatic Remodeling Accompanies Macrophage Accumulation in Superovulation Models

**Figure 3:**
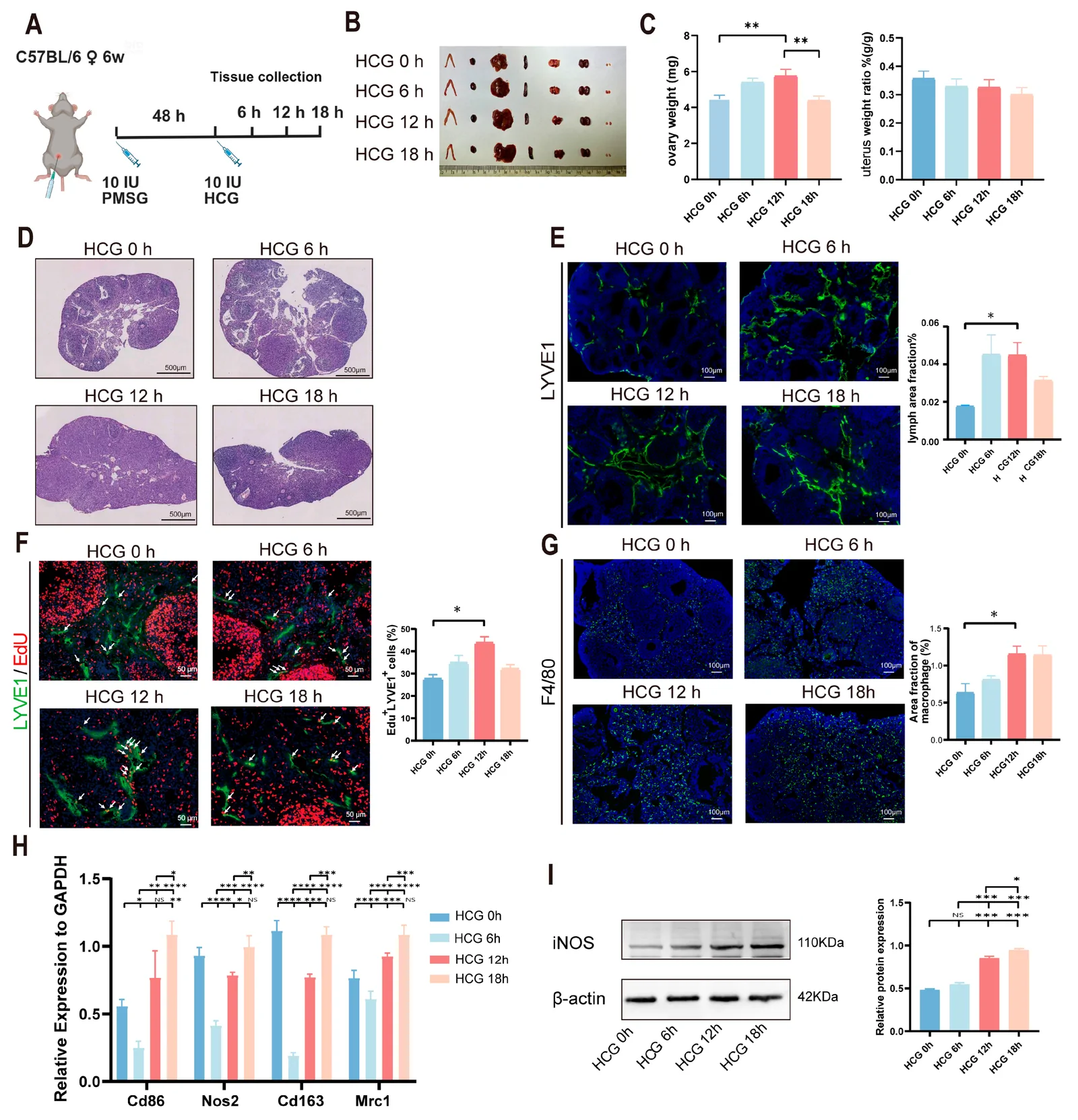
Dynamic lymphatic remodeling accompanies macrophage accumulation during superovulation. (**A**) Schematic diagram of the superovulation protocol in 6-week-old female mice using PMSG followed by hCG. Ovaries were collected at 0, 6, 12, and 18 h after hCG administration. (**B**) Representative images of the ovaries, uterus, and other organs from mice at the indicated time points after hCG administration. (**C**) Ovarian weight (**left**) and organ indices, including the uterus weight-to-body weight ratio (**right**), at 0, 6, 12, and 18 h after hCG administration (*n* = 5). (**D**) Representative hematoxylin and eosin (H&E)-stained ovarian sections at the indicated time points after hCG administration. Scale bars, 500 μm. (**E**) Representative immunofluorescence images of LYVE1^+^ lymphatic vessels in ovarian sections (**left**) and quantification of the LYVE1^+^ lymphatic vessel area fraction (**right**). LYVE1 staining is shown in green. Scale bars, 100 μm (*n* = 5). (**F**) Representative immunofluorescence images of LYVE1 (green) and EdU (red) co-staining in ovarian sections (**left**). Scale bars, 50 μm. White arrows indicate LYVE1^+^EdU^+^ proliferating lymphatic endothelial cells. The proportion of proliferating LECs was quantified as the percentage of EdU^+^ cells among LYVE1^+^ cells (*n* = 5). (**G**) Representative immunofluorescence images of F4/80^+^ macrophages in ovarian sections at the indicated time points (**left**) and quantification of the F4/80^+^ macrophage area fraction (**right**). F4/80 staining is shown in green. Scale bars, 100 μm (*n* = 5). (**H**) Relative mRNA expression levels of macrophage-associated markers Cd86, Nos2, Cd163, and Mrc1 in ovaries at the indicated time points (*n* = 3). (**I**) Representative Western Blot images and quantification of iNOS protein expression in ovaries at the indicated time points (*n* = 3). Data are presented as mean ± SEM. Statistical analyses were performed using one-way ANOVA. NS, not significant, * *p* < 0.05, ** *p* < 0.01, *** *p* < 0.001, **** *p* < 0.0001.

To accurately characterize the dynamic changes in follicular development and corpus luteum formation, we established a superovulation model using pregnant mare serum gonadotropin (PMSG) followed by human chorionic gonadotropin (HCG) in 6-week-old female mice (Figure 3A). Ovarian tissues were collected at defined time points after HCG administration (0 h, 6 h, 12 h, and 18 h), corresponding to distinct stages of follicular development and luteinization. Ovarian weight progressively increased after hCG stimulation, reaching a maximum at 12 h before declining at 18 h (Figure 3B,C). Uterine weight and other organ indices showed no significant changes (Figure 3C and Figure S2A–D). Histological analysis revealed specific morphological changes in the ovary of superovulation model (Figure 3D). At HCG 0 h, ovaries contained numerous large antral follicles with expanded antral cavities. At HCG 6 h, large antral follicles were accompanied by corpus hemorrhagicum and newly formed corpora lutea. At HCG 12 h, ovaries were mainly occupied by multiple well-developed corpora lutea, whereas at HCG 18 h, smaller follicles reappeared in the superficial cortex alongside distinct corpora lutea (Figure 3D).

To examine lymphatic changes during this interval, LYVE1-positive area was low at 0 h, increased at 6 and 12 h, and was lower at 18 h than at the preceding time points (Figure 3E). LYVE1/EdU co-staining showed that the proportion of LYVE1^+^EdU^+^ cells was highest at 12 h after HCG stimulation (Figure 3F). We next assessed macrophage distribution and found that the F4/80-positive area was significantly increased at HCG 12 h compared with HCG 0 h (Figure 3G and Figure S2F,G).

Furthermore, mRNA levels showed that the expression of macrophage associated markers, including *Cd86*, *Nos2*, *Cd163* and *Mrc1*, was increased during the later stages examined (Figure 3H). iNOS protein levels were significantly elevated at HCG 12 h and HCG 18 h (Figure 3I). Together, these findings show that changes in LYVE1-positive lymphatic vessels and associated area occur during the periovulatory and early luteal interval in the superovulation model, with prominent changes observed around 12 h after HCG stimulation.

### 3.4. VEGFR3 Inhibition Impairs Ovarian Lymphangiogenesis and Causes Ovarian Dysfunction

**Figure 4:**
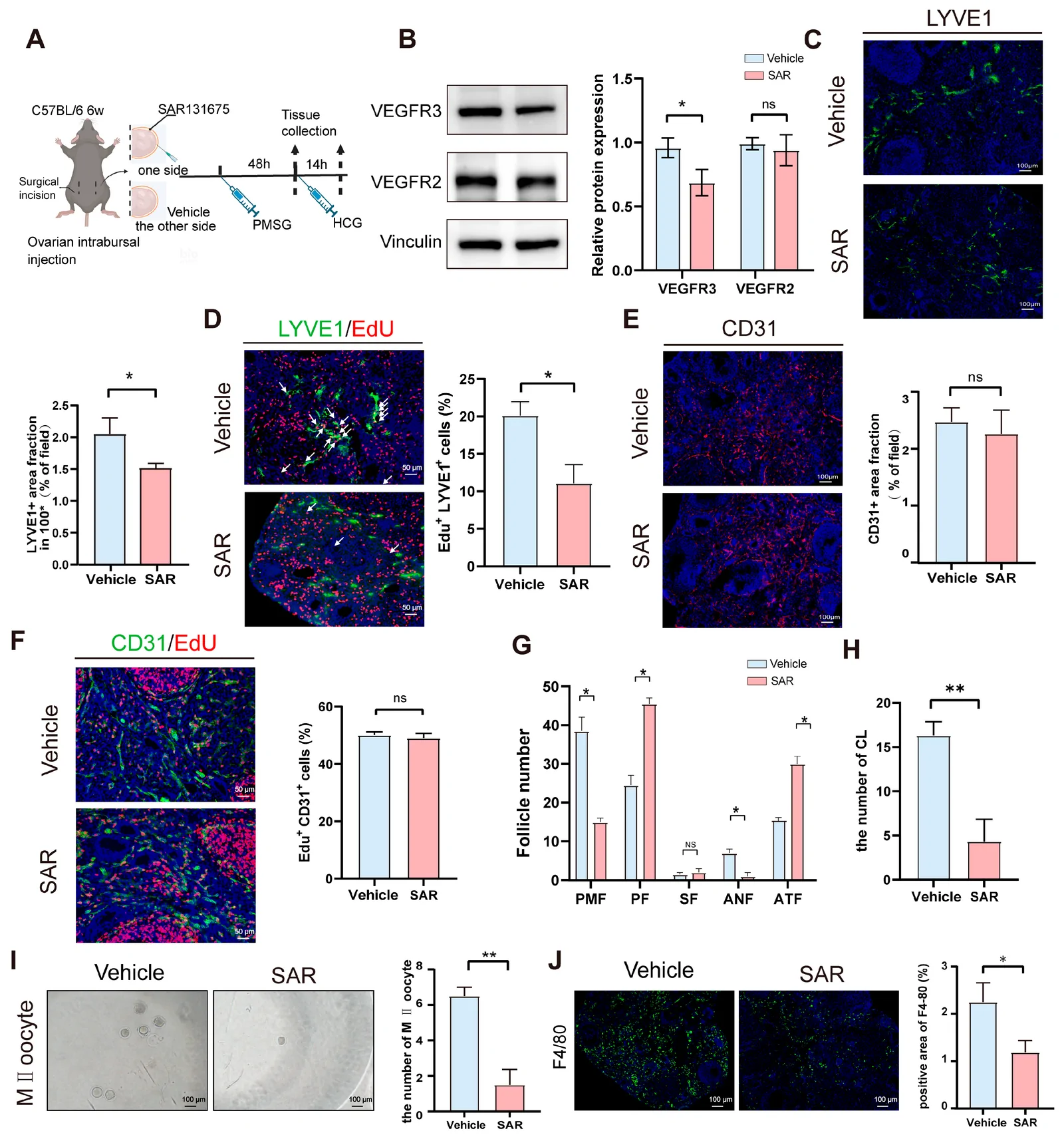
Impaired lymphangiogenesis inhibits follicular development, ovulation, and corpus luteum formation. (**A**) Schematic diagram of unilateral intrabursal injection of SAR131675 (15 μg/ovary) or vehicle. After recovery from anesthesia, mice were treated with PMSG (10 IU) for 48 h. For follicular development analysis, ovaries were collected 48 h after PMSG administration. For ovulation and corpus luteum formation, hCG (10 IU) was administered at 48 h after PMSG, followed by collection of ovaries and MII oocytes 14 h later. (**B**) Representative Western Blot images and quantification of VEGFR3 and VEGFR2 protein expression in ovaries from the control (Con) and SAR131675-treated (SAR) groups (*n* = 3 mice). (**C**) Representative immunofluorescence images of LYVE1^+^ lymphatic vessels in ovarian sections of Vehicle and SAR-treated groups (**left**) and quantification of the LYVE1^+^ lymphatic vessel area fraction (**right**). LYVE1 staining is shown in green. Scale bars, 100 μm, (*n* = 5 mice). (**D**) Representative immunofluorescence images of LYVE1 (green) and EdU (red) co-staining in ovarian sections of Vehicle and SAR-treated groups (**left**). White arrows indicate LYVE1^+^EdU^+^ proliferating LECs. The proportion of proliferating LECs was quantified as the percentage of EdU^+^ among LYVE1^+^ cells. Scale bars, 50 μm, (*n* = 5 mice). (**E**) Representative immunofluorescence images of CD31^+^ blood vessels (**left**) and quantification of the CD31^+^ vessel area fraction (**right**) in ovarian sections of Vehicle and SAR-treated groups. CD31 staining is shown in green. Scale bars, 100 μm, (*n* = 5 mice). (**F**) Representative immunofluorescence images of CD31 (green) and EdU (red) co-staining (**left**) and quantification of CD31^+^EdU^+^ endothelial cells (**right**) in ovarian sections of Vehicle and SAR-treated groups, Scale bars, 50 μm, (*n* = 5 mice). (**G**) Quantification of follicle numbers at different developmental stages, including PMF, PF, SF, ANF, and ATF in ovarian sections of Vehicle and SAR-treated groups, (*n* = 5 mice). (**H**) Quantification of corpus luteum (CL) in ovarian sections of Vehicle and SAR-treated groups, (*n* = 5 mice). (**I**) Representative images of MII oocytes (**left**) and quantification of MII oocyte number (**right**) in Vehicle and SAR-treated groups, Scale bars, 100 μm, (*n* = 5 mice). (**J**) Representative immunofluorescence images of F4/80^+^ macrophages (**left**) and quantification of F4/80^+^ area fraction (**right**) in ovarian sections of Vehicle and SAR groups. F4-80 staining is shown in green. Scale bars, 100 μm, (*n* = 5 mice). NS, not significant, * *p* < 0.05, ** *p* < 0.01.

To investigate the functional role of ovarian lymphatic vessels, we performed unilateral intrabursal administration of the VEGFR3 inhibitor SAR131675, with the contralateral ovary receiving vehicle as a control. After recovery from anesthesia, mice received PMSG (10 IU) for 48 h. For analysis of follicular development, ovaries were collected 48 h after PMSG administration. For assessment of ovulation and corpus luteum formation, hCG (10 IU) was administered 48 h after PMSG, followed by collection of ovaries and MII oocytes 14 h later (Figure 4A).

To verify the inhibitory activity and target specificity of SAR131675 under our experimental conditions, we assessed VEGFR3 expression in vehicle and SAR groups. VEGFR3 protein expression was significantly reduced in SAR groups compared with vehicle groups (Figure 4B). Previous studies have shown that SAR131675 retains moderate inhibitory activity against VEGFR2, particularly at higher concentrations or doses, despite its greater selectivity for VEGFR3 [33]. Under our experimental conditions, SAR131675 treatment did not result in a significant change in ovarian VEGFR2 expression (Figure 4B). VEGFR3 staining was also reduced following SAR131675 treatment, while VEGFR2 staining was not significantly altered (Figure S3B–E). Negative controls are shown in Figure S3F.

We next examined ovarian lymphatic vessels. LYVE1-positive lymphatic vessel area fraction was significantly reduced in SAR groups compared with vehicle groups (Figure 4C). LYVE1^+^EdU^+^ cells were also significantly reduced following SAR131675 treatment (Figure 4D). In contrast, neither CD31-positive vessel area fraction nor CD31^+^ EdU^+^ endothelial cells differed significantly between the SAR131675-treated and vehicle control groups (Figure 4E,F).

We further assessed ovarian function. Ovarian weight did not differ significantly between the paired treatment conditions (Figure S3A). SAR131675 treatment was associated with changes in follicular composition, including reduced numbers of PMF and ANF and increased numbers of PF and atretic follicles (ATF) (Figure 4G). The number of corpora lutea was also significantly reduced following SAR131675 treatment (Figure 4H). The number of MII oocytes was significantly lower in SAR group (Figure 4I). F4/80-positive area was also significantly reduced following SAR131675 treatment (Figure 4J).

Together, SAR131675 treatment selectively is associated with reduced LYVE1-positive lymphatic vessels and lymphatic endothelial cells proliferation, accompanied by impaired follicular development, reduced ovulation and corpus luteum formation, and decreased macrophage accumulation.

### 3.5. Ovarian Lymphatic Vessels Promote Macrophage Accumulation Through the CCL21–CCR7 Signaling Axis

**Figure 5:**
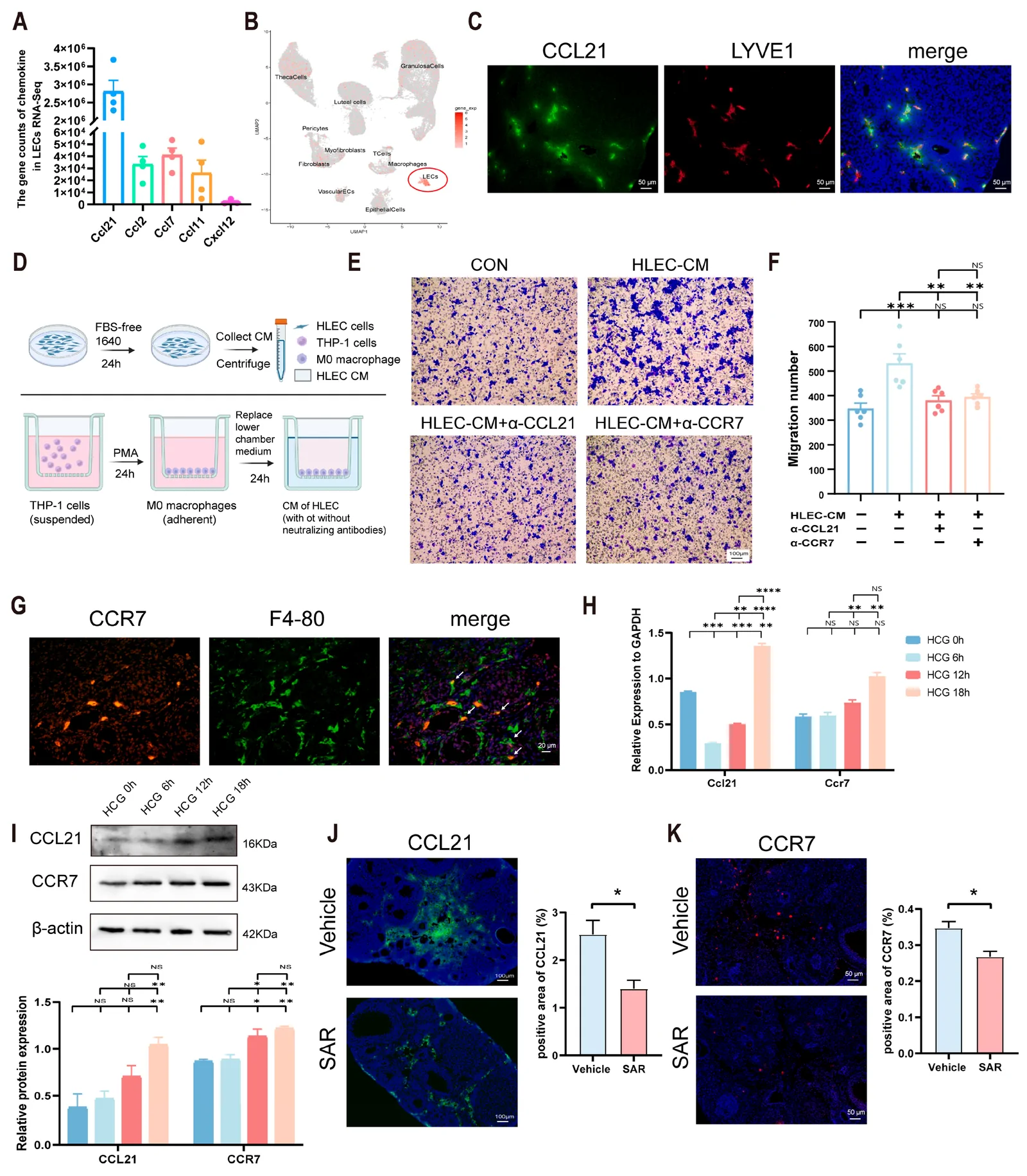
Lymphatic-derived CCL21 contributes to macrophage recruitment during folliculogenesis. (**A**) Transcriptomic profiling of isolated ovarian LECs from young mice showing expression levels of multiple chemokines, with prominent expression of *Ccl21* (*n* = 4). (**B**) UMAP plot of ovarian single-cell RNA sequencing data showing enrichment of CCL21 expression in lymphatic endothelial cell cluster. The circle indicates the lymphatic endothelial cell cluster with enriched *Ccl21* expression. (**C**) Representative immunofluorescence images showing CCL21 expression in LYVE1^+^ ovarian lymphatic vessels. CCL21 staining is shown in green, LYVE1 staining is shown in red, and nuclei are counterstained with DAPI (blue). Scale bars, 50 μm. (**D**) Schematic illustration of the Transwell migration assay using conditioned medium from human LECs to macrophage migration. (**E**) Representative images of migrated THP-1-derived macrophages under indicated treatment conditions, including control medium, HLEC-conditioned medium (HLEC-CM), HLEC-CM, and treatment with CCL21 or CCR7 neutralizing antibodies. Purple staining indicates migrated macrophages. (**F**) Quantification of migrated macrophages in Transwell assays is shown in (**E**) (*n* = 6). (**G**) Representative immunofluorescence images showing co-localization of CCR7 and F4/80 in ovarian section. F4-80 staining is shown in green, CCR7 staining is shown in red, and nuclei are counterstained with DAPI (blue). White arrows indicate CCR7^+^ F4/80^+^ cells, Scale bars, 25 μm. (**H**) qPCR analysis showing dynamic expression changes in Ccl21 and Ccr7 following hCG stimulation at indicated time points. Data were normalized to Gapdh (*n* = 3). (**I**) Representative Western Blot images and quantification of CCL21 and CCR7 protein expression in ovaries at the indicated time points following hCG administration. (**J**) Representative immunofluorescence images and quantification of ovarian CCL21 expression following treatment with Vehicle or the VEGFR3 inhibitor SAR131675. CCL21 staining is shown in green. (**K**) Representative immunofluorescence images and quantification of ovarian CCR7 expression following treatment with Vehicle or SAR131675. CCR7 staining is shown in red. Data are presented as mean ± SEM. * *p* < 0.05, ** *p* < 0.01, *** *p* < 0.001, **** *p* < 0.0001, NS, not significant.

To identify a potential molecular link between ovarian LECs and macrophages, we reanalyzed transcriptomic data from FACS-isolated mouse ovarian LECs. *Ccl21* was prominent among the chemokines examined (Figure 5A). Reanalysis of our previously generated human ovarian single-cell RNA sequencing dataset [34] further demonstrated that CCL21 expression was highly enriched in the LECs cluster (Figure 5B). Consistent with these findings, CCL21 staining was detected in LYVE1-positive lymphatic vessels in ovarian tissue (Figure 5C). CCL21 staining was also detected in both human and mouse lymphatic endothelial cells, while its receptor, CCR7, was detected in both human THP 1-derived macrophages and mice RAW264.7 macrophages (Figure S4A,B).

We next examined whether LECs-derived CCL21 contributes to macrophage recruitment. Conditioned medium from human lymphatic endothelial cells (HLEC CM) was collected and applied to Transwell migration assays using THP-1-derived macrophages (Figure 5D). HLEC CM significantly increased macrophage migration compared with control medium (Figure 5E,F). Neutralization of either CCL21 or its receptor CCR7 significantly attenuated HLEC CM induced macrophage migration (Figure 5E,F). Control experiments showed that the CCL21 and CCR7 neutralizing antibodies alone did not significantly affect macrophage migration in the absence of HLEC CM (Figure S4C,D), these results support a contribution of CCL21–CCR7 signaling to the migratory response. Consistent findings were obtained using the mouse lymphatic endothelial cell line SVEC4-10 and mouse macrophage cell line RAW264.7. Conditioned medium from SVEC4-10 cells significantly increased RAW264.7 macrophage migration, whereas neutralization of either CCL21 or its receptor CCR7 significantly attenuated this migratory response. Control experiments confirmed that the neutralizing antibodies alone did not significantly affect macrophage migration in the absence of SVEC4-10 conditioned medium (Figure S4E,F).

We next examined whether ovarian macrophages express CCR7 and may therefore respond to lymphatic derived CCL21. CCR7 staining partially overlapped with F4/80-positive macrophages in ovarian tissue (Figure 5G). *Ccl21* transcript abundance decreased at 6 h, remained below the 0 h level at 12 h, and was highest at 18 h, whereas *Ccr7* expression increased later in the time course (Figure 5H). Ovarian CCL21 and CCR7 protein abundance was also higher at the later time points after hCG injection (Figure 5I). These findings indicate that the CCL21–CCR7 signaling components are dynamically regulated during the periovulatory and early luteal interval.

Finally, we examined whether VEGFR3 inhibition altered this signaling axis in vivo. Local SAR131675 treatment reduced ovarian CCL21 expression and was accompanied by decreased CCR7-positive signals in ovarian tissue (Figure 5J,K). Together, these results indicate that lymphatic-derived CCL21–CCR7 signaling regulates macrophage migration, which in turn may modulate ovarian function.

## 4. Discussion

Ovarian lymphatic vessels are hormonally regulated and have been implicated in reproductive function [10,35,36], while recent evidence that lymphatic endothelial cells can influence macrophage behavior raises the possibility of a local lymphatic–immune interaction [16]. Whether such interactions contribute to physiological ovarian remodeling, however, remains unclear. Here, we demonstrate that ovarian lymphatic remodeling accompanies changes in macrophage abundance and distribution across follicular stages, the estrous cycle, and a gonadotropin-induced periovulatory time course, while pharmacological VEGFR3 inhibition reduces lymphatic expansion and macrophage accumulation and impairs reproductive outcomes. Our findings identify ovarian lymphatic remodeling as a component of reproductive tissue remodeling associated with macrophage dynamics and further support a role for CCL21–CCR7 signaling in LEC-conditioned medium-induced macrophage migration in vitro.

Lymphatic remodeling was evident across follicular development, the natural estrous cycle, and the gonadotropin-induced periovulatory course. During follicular development, LYVE1-positive area and the proportion of EdU-positive cells among LYVE1-positive cells increased, with prominent lymphatic structures around corpora lutea. In naturally cycling ovaries, lymphatic vessel number and area fraction, Lyve1 transcript abundance, and VEGFC-positive area were highest at diestrus. In the superovulation model, LYVE1-positive area increased at 6 and 12 h after hCG, whereas the proportion of LYVE1/EdU double-positive cells peaked at 12 h around the time of ovulation. These complementary models show that ovarian lymphatic remodeling varies with reproductive stage and hormonal context. Because the naturally cycling and superovulation cohorts differed in age and endocrine treatment, their response magnitudes should not be compared directly. The observed changes extend previous evidence that ovarian lymphatic growth is hormonally regulated [9]. Three-dimensional imaging and lineage tracing have shown that ovarian blood vessels undergo extensive angiogenic remodeling around follicles and corpora lutea, establishing transient vascular networks during follicular development [4]. Our findings extend this vascular-remodeling framework by demonstrating stage-dependent changes in the ovarian lymphatic network alongside the established remodeling of ovarian blood vessels.

Local SAR131675 treatment reduced ovarian VEGFR3 expression, LYVE1-positive area, and LYVE1/EdU double-positive cells, while VEGFR2 expression and the measured CD31-positive vascular parameters remained unchanged. These findings confirm target-associated changes in the ovarian lymphatic compartment without detectable alterations in the blood vasculature. SAR131675 treatment also altered follicular composition, with fewer primordial and antral follicles and more primary and atretic follicles, and reduced the numbers of corpora lutea and recovered MII oocytes. These findings link local VEGFR3 inhibition and reduced lymphatic expansion with changes in follicular composition, luteal formation, and ovulatory output. In addition, F4/80-positive area was also lower in treated ovaries, suggesting that lymphatic vessels may affect ovarian function through modulation of the ovarian immune microenvironment. Because the intervention was pharmacological, these data support a functional contribution of VEGFR3-sensitive ovarian remodeling but do not by themselves establish that each reproductive effect is mediated exclusively by lymphatic vessels. Future studies using genetic models (e.g., lymphatic-specific Vegfr3 deletion) are needed to establish whether the observed reproductive effects are causally mediated by lymphatic vessels.

Macrophages are important components of the ovarian microenvironment, contributing to vascular integrity, follicular development, and tissue remodeling [37,38]. Although their roles in ovarian physiology are increasingly recognized, the local mechanisms coordinating macrophage distribution during follicular development and ovulation remain incompletely understood. The accompanying changes in F4/80-positive area suggest that lymphatic remodeling is associated with the maintenance or redistribution of ovarian macrophages, demonstrating that ovarian macrophage abundance changes alongside the lymphatic network. Recent studies further support a role for macrophages in follicular development. In a mouse ovary co-culture model, macrophages promoted primordial follicle activation [39], whereas in a high-fat-diet model, ovarian macrophage infiltration and proinflammatory cytokine production were associated with excessive primordial follicle activation [40]. Experimental macrophage depletion has also been shown to disrupt ovarian vascular integrity, increase follicular atresia, and impair ovulation [41,42]. In the present study, local VEGFR3 inhibition reduced macrophage accumulation and was accompanied by increased atretic follicle numbers, fewer corpora lutea, and reduced MII-oocyte recovery. Together, these findings support an association between ovarian lymphatic remodeling, macrophage accumulation, and reproductive outcomes. Whether macrophage changes contribute to the reproductive phenotypes following VEGFR3 inhibition remains to be determined.

Our transcriptomic analyses identify CCL21 as a prominent chemokine in mouse ovarian LECs, and reanalysis of the human ovarian single-cell dataset showed CCL21 enrichment in the LEC cluster [34]. CCL21 immunoreactivity in LYVE1-positive ovarian vessels further supports LECs as a local source, while partial overlap of CCR7 and F4/80 staining identifies a potentially responsive macrophage subset. Functional assays using both human and mouse cell systems showed that lymphatic endothelial cell-conditioned media enhanced macrophage migration and that neutralization of either CCL21 or CCR7 attenuated this response. The lack of an effect of either antibody in the absence of conditioned medium supports the interpretation that the reduced migration was associated with blockade of the conditioned-medium-induced response. These findings are consistent with reports that LEC-derived mediators regulate macrophage behavior in other settings, including decidual LEC-derived GM-CSF in pre-eclampsia [16], and with studies implicating CCL21–CCR7 signaling in macrophage migration in inflammatory and tumor models [43,44,45]. CCL21 has also been reported to promote porcine oocyte maturation in vitro [46,47], indicating that its ovarian actions may extend beyond immune regulation. However, whether CCL21–CCR7 signaling directly regulates ovarian function, and whether its effects on oocyte maturation are conserved in the human ovary, remain to be determined and warrant further investigation.

The periovulatory time course further showed that ovarian Ccl21 and Ccr7 expression changed over time, with Ccl21 transcript abundance lowest at 6–12 h and highest at 18 h after hCG, while Ccr7 expression increased at later time points. CCL21 and CCR7 protein abundance were also higher at the later time points, and VEGFR3 inhibition reduced ovarian CCL21 and CCR7-positive signals. These temporal changes do not fully parallel the peak in lymphatic area and LYVE1/EdU labeling at 12 h, indicating that lymphatic expansion and chemokine–receptor expression are temporally related but not synchronous. CCL21 activity also depends on its spatial presentation and extracellular processing, which regulate immune-cell entry into lymphatics [48,49]. Thus, tissue staining and macrophage proximity to lymphatic vessels do not demonstrate a functional chemokine gradient or distinguish macrophage recruitment from retention or egress. Although the agreement between human and mouse cell-line assays strengthens the in vitro evidence, neither system reproduces the interactions between primary ovarian LECs and resident macrophages. Cell-specific manipulation of Ccl21 or Ccr7 and direct in vivo tracking of macrophages will be required to establish the causal contribution of this pathway to ovarian macrophage dynamics and reproductive function.

## 5. Conclusions

**Figure 6:**
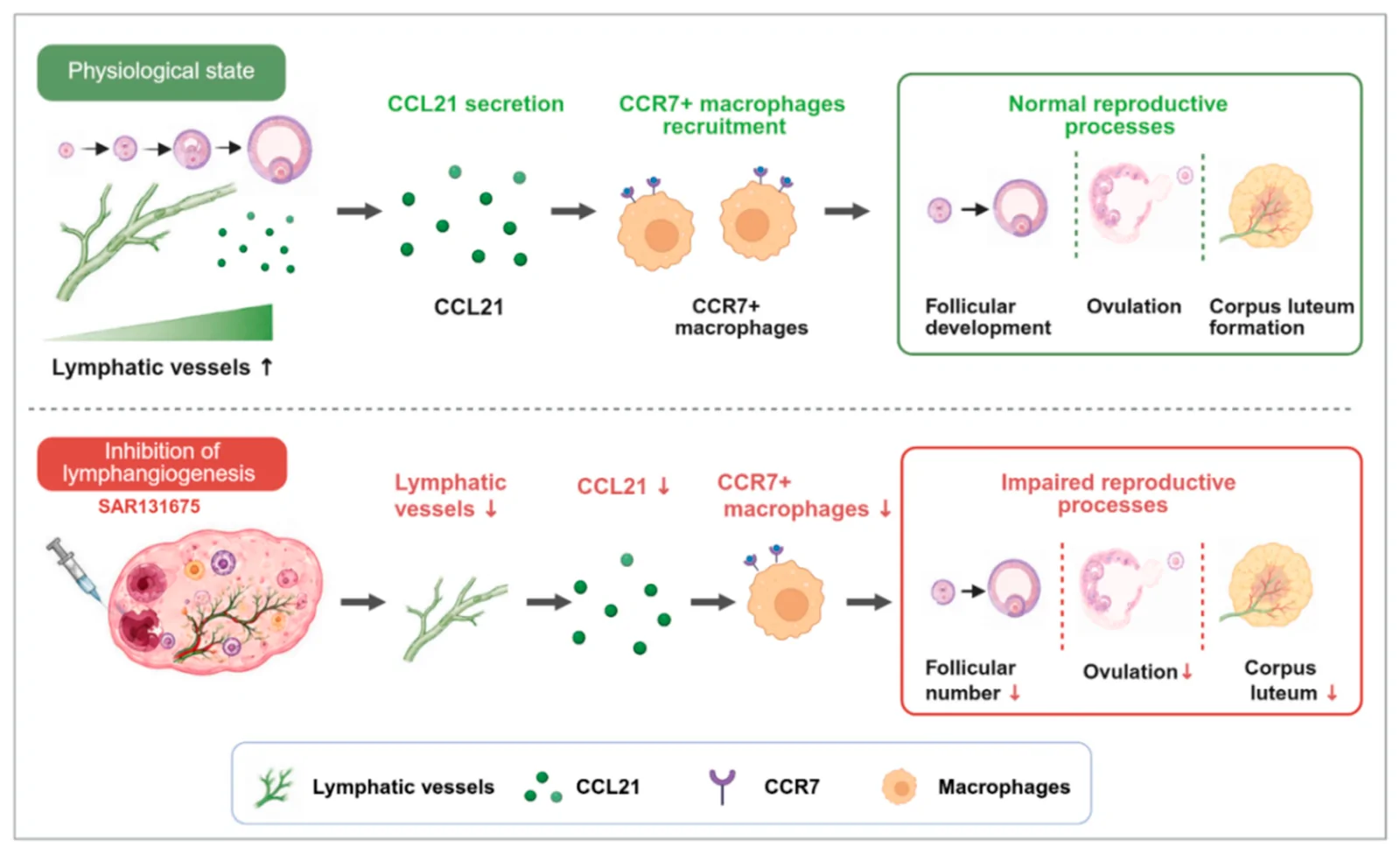
Ovarian lymphatic vessels undergo dynamic remodeling during follicular development and contribute to ovarian reproductive function by regulating macrophage recruitment through CCL21–CCR7 signaling. Downward arrows (↓) indicate a decrease in the corresponding parameters.

This study demonstrates that ovarian lymphatic vessels undergo dynamic remodeling during cyclical ovarian changes, accompanied by cyclic changes in macrophage abundance. Disruption of ovarian lymphangiogenesis reduces macrophage accumulation and is associated with impaired follicular development, corpus luteum formation, and ovulation. Our findings further support a role for the CCL21–CCR7 signaling axis in mediating communication between ovarian lymphatic endothelial cells and macrophages. Collectively, these findings identify ovarian lymphatic vessels as an important component of the ovarian immune microenvironment and suggest that lymphatic–macrophage interactions contribute to the regulation of ovarian function (Figure 6).

## Figures and Tables

**Table 1:** Primer sequences used for quantitative real-time PCR (qRT-PCR).

Genes	Primers	Sequence (5′–3′)
*Gapdh*	Forward primer	TGACCTCAACTACATGGTCTACA
	Reverse primer	CTTCCCATTCTCGGCCTTG
*L* *yve1*	Forward primer	AGGTAGAGTCAGCGCAGAAAT
	Reverse primer	AGAAAATCCGAGGGATGACAGA
*C* *d86*	Forward primer	TCAATGGGACTGCATATCTGCC
	Reverse primer	GCCAAAATACTACCAGCTCACT
*N* *os2*	Forward primer	GATGTTGAACTATGTCCTATCTCC
	Reverse primer	GAACACCACTTTCACCAAGAC
*C* *d163*	Forward primer	GGTGGACACAGAATGGTTCTTC
	Reverse primer	CCAGGAGCGTTAGTGACAGC
*Mrc1*	Forward primer	CTCTGTTCAGCTATTGGACGC
	Reverse primer	TGGCACTCCCAAACATAATTTGA
*C* *cl21*	Forward primer	GTGATGGAGGGGGTCAGGA
	Reverse primer	GGGATGGGACAGCCTAAACT
*C* *cr7*	Forward primer	GCCCAGATGGTTTTTGGGTTC
	Reverse primer	GCAAGGTACGGATGATAATGAGG

## Data Availability

The datasets utilized and examined in this research are accessible upon request from the corresponding author.

## Supplementary Materials

The following supporting information can be downloaded at https://www.mdpi.com/article/10.3390/biom16101499/s1. Figure S1. Ovarian lymphatic vessels and macrophages during the estrous cycle, with immunohistochemical and edu negative controls. Figure S2. Organ indices and ovarian macrophage distribution following hCG stimulation. Figure S3. Ovarian VEGFR3 and VEGFR2 expression following SAR131675 treatment. Figure S4. Expression of CCL21 and CCR7 and validation of CCL21-CCR7-dependent macrophage migration in vitro. Figure S5. Original Western blot images corresponding to Figure 3I. (A) iNOS. (B) β-actin. Figure S6. Original Western Blot images corresponding to Figure 4B, showing VEGFR3, VEGFR2, and vinculin. Figure S7. Original Western Blot images corresponding to Figure 5I. (A) CCR7. (B) β-actin. (C) CCL21. Additional original blot images showing β-actin as the loading control are also provided.

## Abbreviations

The following abbreviations are used in this manuscript:

CCL21	C-C motif chemokine ligand 21
CCR7	C-C motif chemokine receptor 7
VEGFR3	Vascular endothelial growth factor receptor 3
LYVE1	Lymphatic vessel endothelial hyaluronan receptor 1
PMF	Primordial follicles
PF	Primary follicles
SF	Secondary follicles
ANF	Antral follicles
POF	Preovulatory follicles
CL	Corpora lutea
