# Supplementary material for: Dynamic Ovarian Lymphatic Remodeling Is Associated with Macrophage Accumulation During Follicular Development and Ovulation in Mice
Source: Biomolecules. 2026 Oct 15;16(10):1499. doi: 10.3390/biom16101499 (PMC13702732; doi:10.3390/biom16101499)
Supplement: Supplementary file 1 [file biomolecules-16-01499-s001.zip › biomolecules-4550909-supplementary.pdf]

Supplementary material

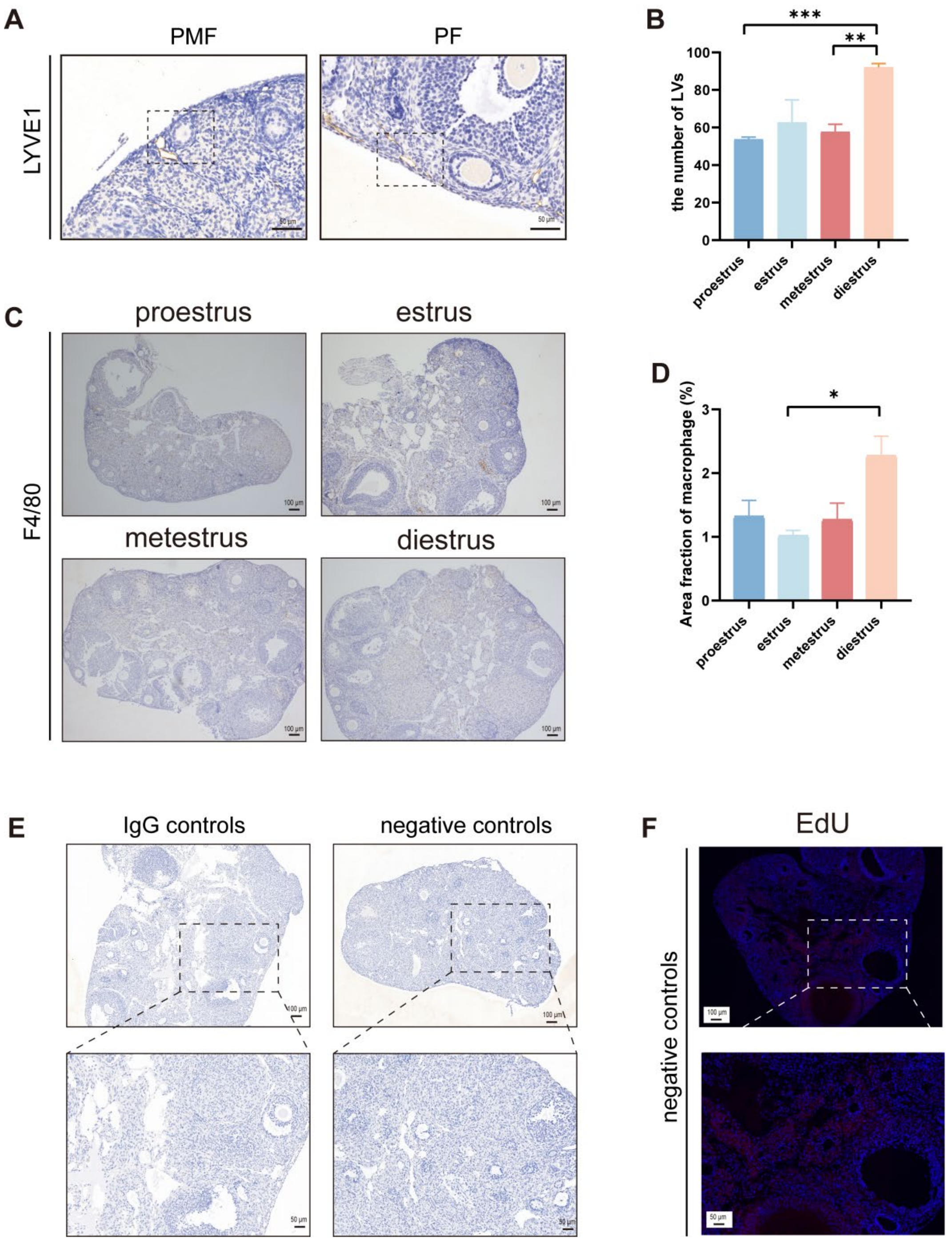

Figure S1. Ovarian lymphatic vessels and macrophages during the estrous cycle, with immunohistochemical and edu negative controls. (A) Representative immunohistochemical images showing LYVE1$^+$ lymphatic vessels surrounding primordial and primary follicles in mouse ovarian sections. Scale bars, 50 μm. (B) Quantification of the number of LYVE1$^+$ lymphatic vessels at

different estrous cycle stages (n = 6). (C) Representative immunohistochemical images of F4/80$^+$ macrophages in mouse ovarian sections at different estrous cycle stages. Scale bars, 100 µm. (D) Quantification of the F4/80$^+$ macrophage area fraction at different estrous cycle stages (n = 6). (E) Representative IgG control and negative control images for VEGFC and F4/80 immunohistochemical staining in ovarian sections. (F) Magnified views corresponding to the upper and lower panels. Scale bars, 100 µm (upper panels, 20× magnification) and 50 µm (lower panels, 40× magnification). NS, not significant, * p < 0.05, ** p < 0.01, *** p < 0.001.

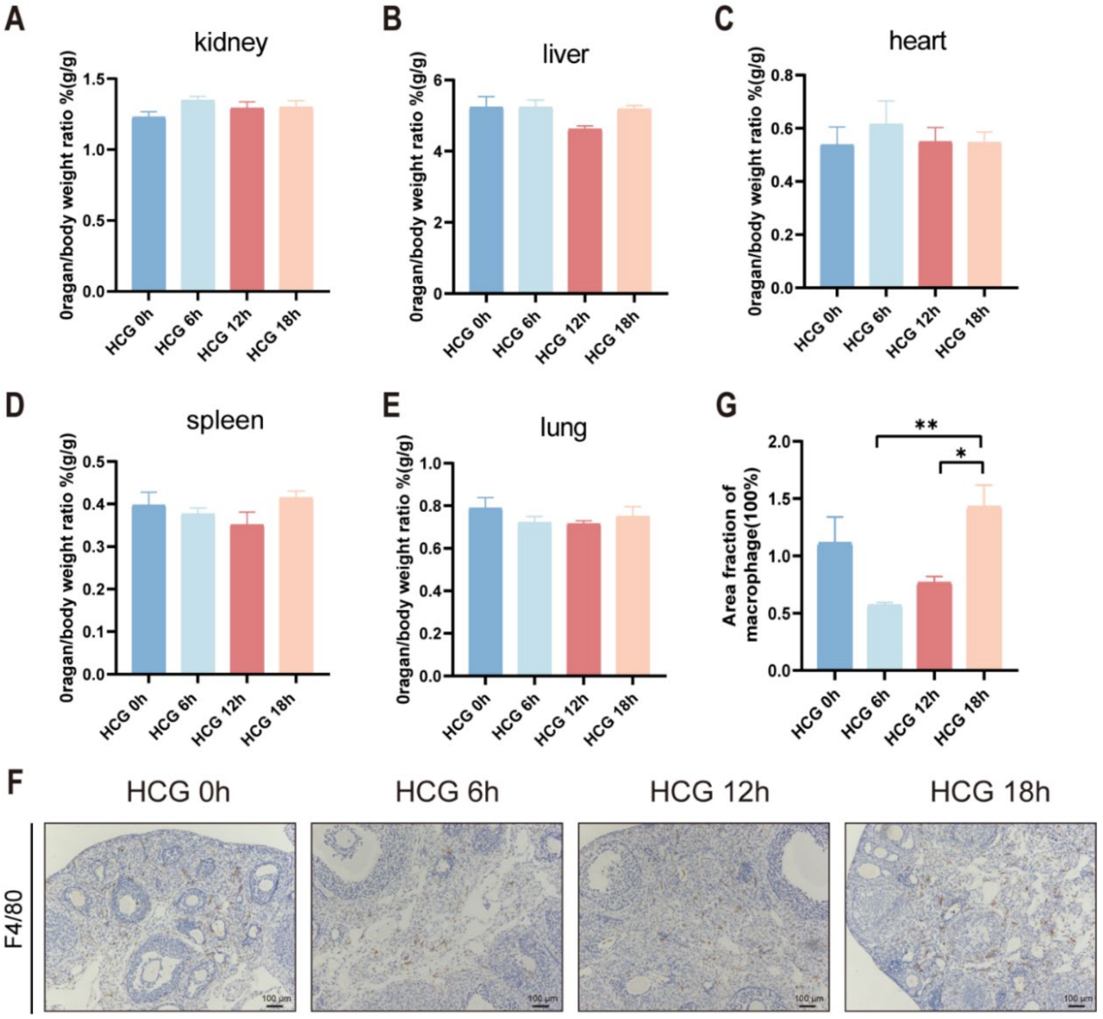

Figure S2. Organ indices and ovarian macrophage distribution following hCG stimulation. (A–E) Organ indices (organ weight-to-body weight ratios; right panels) at 0, 6, 12, and 18 h after hCG stimulation (n = 5). (F) Representative immunohistochemical images of F4/80$^+$ macrophages in

ovarian sections at 0, 6, 12, and 18 h after hCG stimulation. Scale bars, 100 μm. (G) Quantification of the F4/80^{+} macrophage area fraction at the indicated time points after hCG stimulation (n = 6). NS, not significant, * p < 0.05, ** p < 0.01.

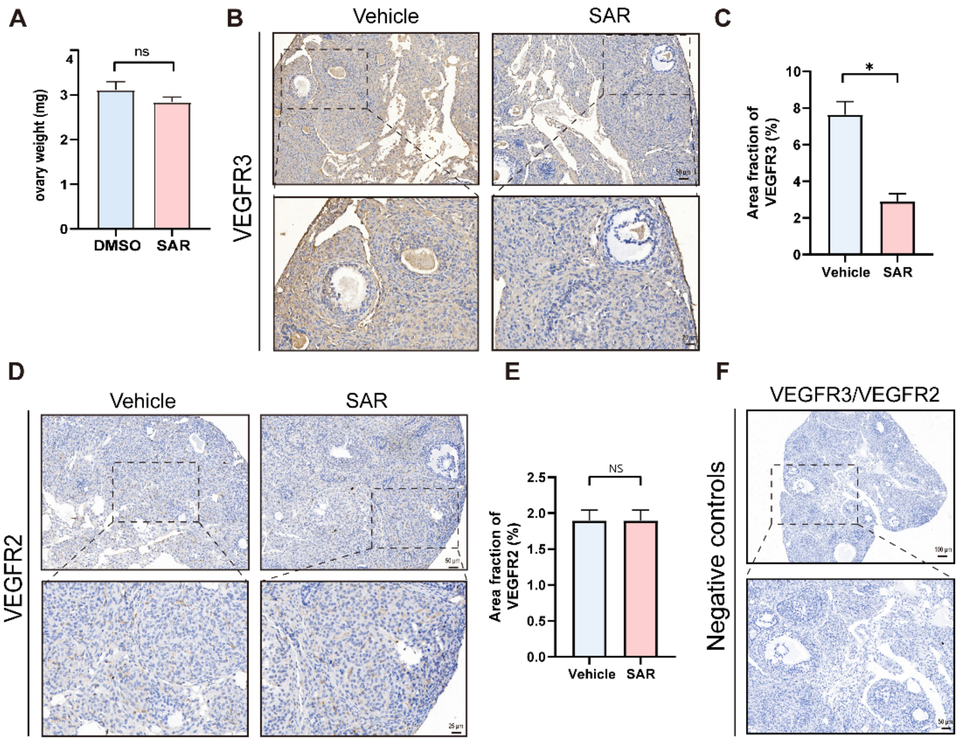

Figure S3. Ovarian VEGFR3 and VEGFR2 expression following SAR131675 treatment. (A) Representative immunohistochemical images and quantification of VEGFR3 expression in ovarian sections from the vehicle and SAR131675-treated groups. (B) Representative immunohistochemical images and quantification of VEGFR2 expression in ovarian sections from the vehicle and SAR131675-treated groups (n = 5). Scale bars, 50 μm (upper panels, 20× magnification) and 25 μm (lower panels, 40× magnification). NS, not significant, * p < 0.05.

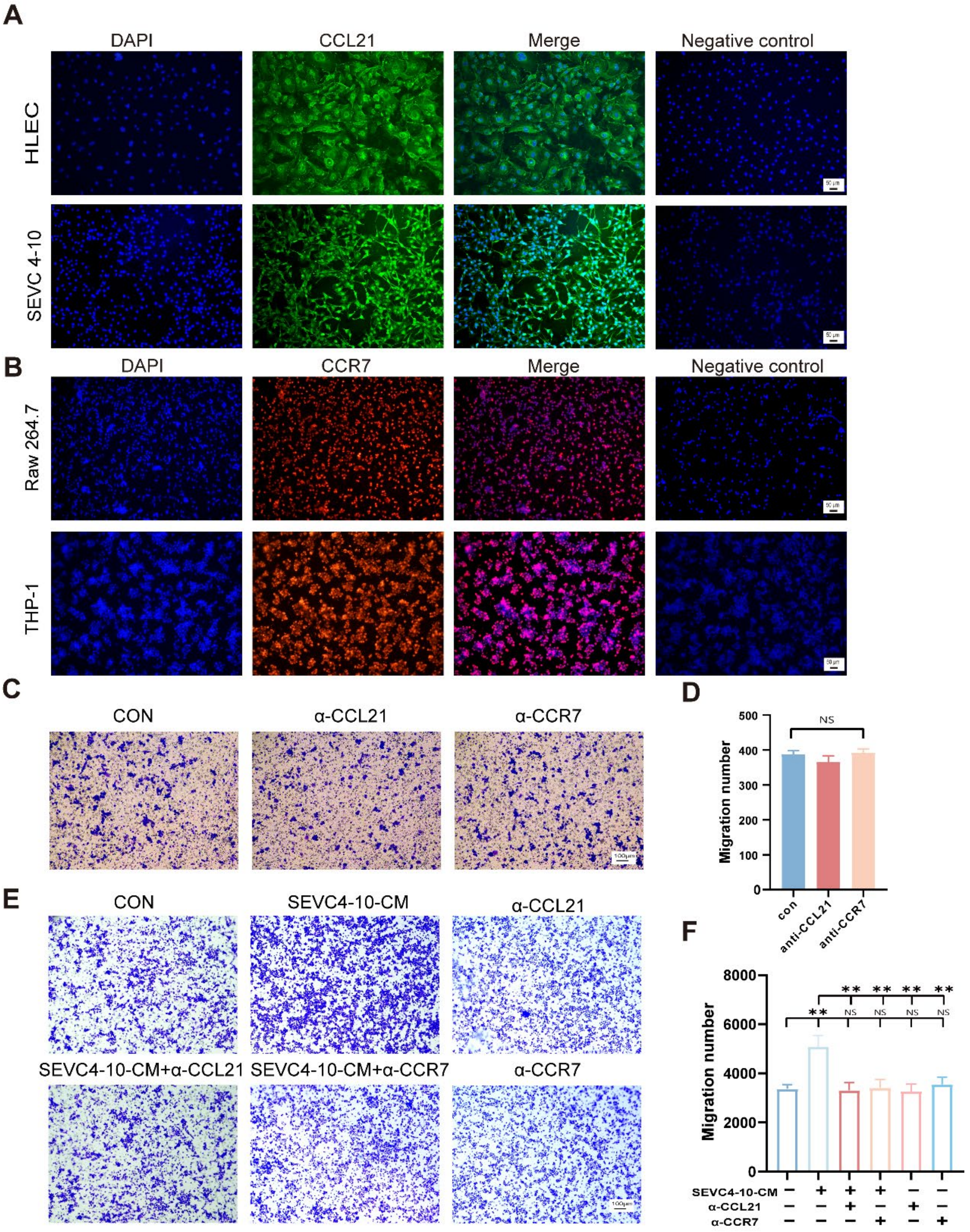

Figure S4. Expression of CCL21 and CCR7 and validation of CCL21-CCR7-dependent macrophage migration in vitro. (A) CCL21 immunostaining in HLECs and SVEC4-10 cells. (B) CCR7 immunostaining in THP-1-derived macrophages and RAW264.7 cells. Scale bars, 50 µm. (C, D) Transwell migration assays of THP-1-derived macrophages following treatment with CCL21-neutralizing or CCR7-neutralizing antibodies, together with quantification of migrated cells. Scale

bars, 100 µm. (E, F) Transwell migration assays of RAW264.7 macrophages exposed to conditioned medium from SVEC4-10 cells in the presence or absence of CCL21-neutralizing or CCR7-neutralizing antibodies, together with quantification of migrated cells. Scale bars, 100 µm (n = 5). NS, not significant, * $p < 0.05$, ** $p < 0.01$.

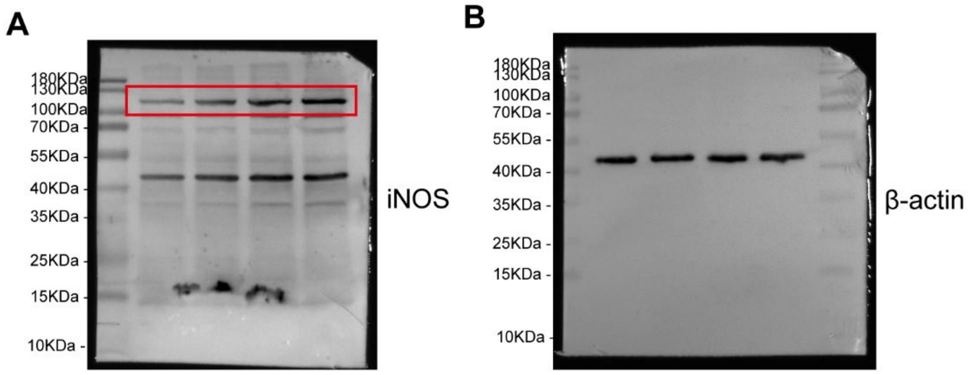

Figure S5 Original Western blot images corresponding to Figure 3I. (A) iNOS. (B) β-actin.

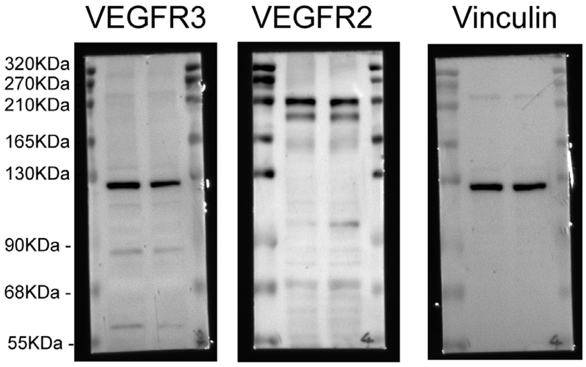

Figure S6 Original Western Blot images corresponding to Figure 4B, showing VEGFR3, VEGFR2, and vinculin.

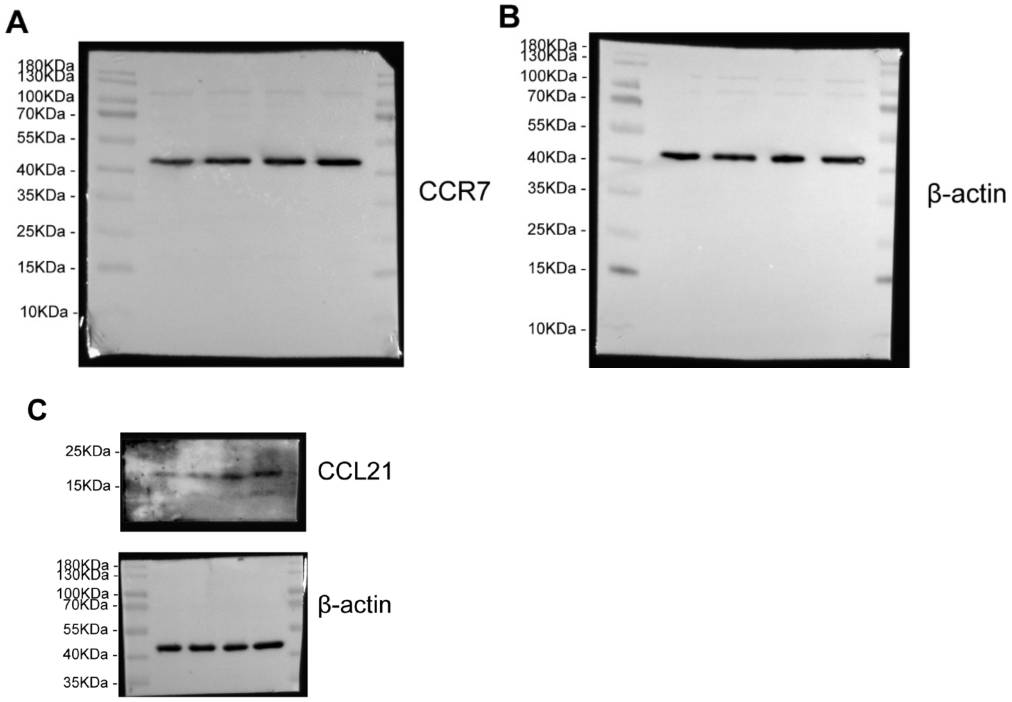

Figure S7 Original Western Blot images corresponding to Figure 5I. (A) CCR7. (B) β-actin. (C) CCL21. Additional original blot images showing β-actin as the loading control are also provided.
